# An Adaptive Measurement System for the Simultaneous Evaluation of Frequency Shift and Series Resistance of QCM in Liquid

**DOI:** 10.3390/s21030678

**Published:** 2021-01-20

**Authors:** Ada Fort, Enza Panzardi, Valerio Vignoli, Marco Tani, Elia Landi, Marco Mugnaini, Pietro Vaccarella

**Affiliations:** Department of Information Engineering and Mathematical Sciences, University of Siena, 53100 Siena, Italy; panzardi@diism.unisi.it (E.P.); valerio.vignoli@unisi.it (V.V.); tani@diism.unisi.it (M.T.); landi@diism.unisi.it (E.L.); marco.mugnaini@unisi.it (M.M.); pietrovaccarella@gmail.com (P.V.)

**Keywords:** QCM sensors, in-liquid measurements, bridge oscillators

## Abstract

In this paper, a novel measurement system based on Quartz Crystal Microbalances is presented. The proposed solution was conceived specifically to overcome the measurement problems related to Quartz Crystal Microbalance (QCM) applications in dielectric liquids where the Q-factor of the resonant system is severely reduced with respect to in-gas applications. The QCM is placed in a Meacham oscillator embedding an amplifier with adjustable gain, an automatic strategy for gain tuning allows for maintaining the oscillator frequency close to the series resonance frequency of the quartz, which is related in a simple way with the physical parameters of interest. The proposed system can be used to monitor simultaneously both the series resonant frequency and the equivalent electromechanical resistance of the quartz. The feasibility and the performance of the proposed method are proven by means of measurements obtained with a prototype based on a 10-MHz AT-cut quartz.

## 1. Introduction

In-liquid measurements with quartz-based sensors are used in many different application fields, among which are chemical sensing, biosensing and viscosity measurements [1,2,3,4].

Sensing systems based on quartzes exploit their electromechanical resonant behavior: their piezoelectric properties provide a transduction of mechanical quantities into electrical quantities, whereas the pure elastic behavior of the quartz, which is an almost ideal resonator, is extremely sensitive to the changes of mechanical properties (mass, stiffness and damping). These particular properties have been exploited to obtain many different sensors: the most traditional ones are Quartz Crystal Microbalances (QCM), operating as mass sensors with resolutions down to a few nanograms [5,6,7,8], although, if used in liquid environments, it is possible to employ resonant measurement systems to characterize liquid viscosity and density. In this contest, the use of resonant structures is widely diffused and spread in different applications. Just to mention a couple, García-Arribas et al. developed a magnetoelastic sensor to sensitively measure the viscosity of fluids, aimed at developing an online and real-time monitoring of the lubricant oil degradation in machinery [9], and Zang et al. used a self-oscillating tuning fork device to perform fast measurements of viscosity and density, with the aim of characterizing different phases of liquids in a multiphase flow [10]. Moreover, in several applications, the research interest is focused on the realization of distributed sensor networks using sensor fusion techniques [11,12,13].

The basic structure of a quartz-based chemical or biosensor is the QCM, which is usually an AT-cut quartz disk operating in the MHz range provided by two electrodes on the two opposite surfaces. When the quartz vibrates in air, it is possible to assume no damping, and the modal frequencies of the device are set by the disk thickness [14]. In particular, it can be written [15]:

(1) $$f_{s}{}_{N}\approx N\frac{1}{2t}\sqrt{\frac{\mu_{Q}}{\rho_{Q}}}$$

where $f_{s}{}_{N}$ represents the *N*-th modal frequency, N∈ℵ and *N* odd and *t* is the thickness of the quartz, whereas $\rho_{Q}$ and $\mu_{Q}$ are the quartz density and shear modulus.

A mass sensor is obtained by functionalizing one of the quartz surfaces with a material providing the selective adsorption of a target species. If, due to functionalization and to adsorption, a small additional mass (Δm) is deposited on one of the quartz surfaces, by assuming that the mechanical properties of the added layer are the same as those of the quartz, then the effect changes the resonator thickness and, accordingly, shifts the modal frequencies of a quantity ($\Delta f_{sN}$) related to the added mass:

(2) $$\Delta f_{sN}=-f_{sN}\frac{\Delta t}{t}=-f_{sN}\frac{\Delta m}{m_{Q}}$$

where $m_{Q}$ is the quartz mass, and the linear relation stems from the assumption of small thickness variations. Notice that Equation (2), written in a different form, is also known as the Sauerbrey equation [16].

By means of the piezoelectric effect, the mechanical vibrations are converted into an electrical charge or voltage signal. Therefore, a QCM is, in principle, a linear mass sensor providing the frequency as an output quantity. A typical 10-MHz AT-cut QCM provides, theoretically, a response of $\Delta f_{s1}\left(Hz\right)=-0.8\;\Delta m$ (ng) [17].

A complete sensing system can be obtained by inserting the quartz into an electronic circuit providing the frequency metering [1].

Indeed, the measurement problem is far more complex [1,18]. First of all, the electromechanical vibrations can shift frequency not only due to the added mass (desired effect) but, also, due to the unwanted mechanical damping caused by the added layer, which has usually different and worse mechanical properties with respect to the quartz, especially in biosensors, where it consists of a lossy viscoelastic functionalization film and of the adsorbed target species (biological targets). Moreover, and more importantly, differently from AT-cut quartz oscillating in the air, where the surrounding medium can be well-described as an ideal fluid, and the standing elastic wave causing the vibration is confined in the solid disk, for in-liquid sensors, the surrounding medium is better described by a Newtonian fluid. In this case, part of the shear vibration can be transferred to the liquid and part of the elastic energy dissipated. The interaction with the fluid is, in this case, a non-negligible mechanical load (with both dissipative and conservative characteristics) that causes very large frequency shifts (up to hundreds of ppms). Due to this fact, some sensing systems exploit the dependency of the modal frequency of the quartz on the characteristics of the surrounding fluid to obtain viscosity measurements [14,19,20]. 

As a consequence, QCM-based measurement systems operating in liquid often couple the modal frequency shift assessment with the evaluation of the viscoelastic characteristics of the added layer through the monitoring of other dynamic parameters, such as the dissipation coefficient *δ* or, equivalently, the Q-factor [21,22]. This could unravel the inertial and dissipative behaviour of the ad-layer and enable distinguishing between the added mass and the surrounding liquid effects.

To continue with the issues related to the QCM-based measurements, it must be underlined that the way in which the vibrations are generated and measured can influence their frequency, which can be different from the searched modal frequency: for instance, the front-end circuit loads and influences the electromechanical resonance [1].

In a typical QCM system, the quartz is embedded in the feedback network of an oscillator circuit, and its output frequency is measured by, e.g., a digital frequency meter. QCM systems with dissipation monitoring (D-QCM) also provide a measurement of the dissipation factor *δ* (equivalent to the Q-factor) by inducing transients, e.g., disconnecting the feedback loop [23] and evaluating the transient decay duration. This allows for assessing the damping coefficient of the resonant electromechanical system.

In this paper, the problems related to measurements based on QCM in in-liquid applications are summarized, and a novel system based on a Meacham oscillator embedding an amplifier with adjustable gain is proposed. An automatic strategy is described that allows for gain tuning and maintaining the oscillator frequency close to the modal resonance of the quartz. From the adjusted gain value, an estimation of the series electromechanical resistance can be obtained, providing also the monitoring of the dissipative behavior of the electromechanical system through a robust estimation technique. 

This paper is organized as follows: in Section 2, the operation of quartzes in liquid is presented, and the main measurement problems are discussed. In Section 3, the proposed oscillator topology, the series resistance estimation technique and the gain adjustment strategy are described. In Section 4, a prototype system, used to verify the proposed solution, is described, whereas Section 5 shows and discusses the experimental results. The conclusions are drawn in Section 6.

## 2. In Liquid Measurements Based on QCM and Oscillators

**Figure 1:**
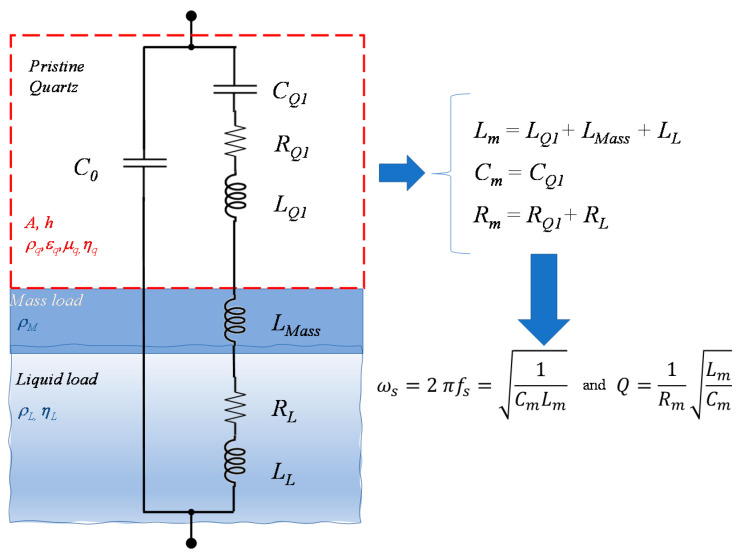
Equivalent lumped parameter circuit for a Quartz Crystal Microbalance (QCM) in in-liquid applications.

In-liquid quartz resonators can be used either to assess the viscoelastic properties of a liquid or to measure the mass of a target biological species. In the latter case, a functionalization layer is deposited over one of the quartz surfaces, ensuring the selective adsorption of the target. The target species are usually dispersed in a liquid; therefore, in both cases, the pristine or functionalized surface of the quartz is in contact with a liquid medium. A general model for this kind of measurement set-up is represented in Figure 1, where the lumped parameter electrical equivalent circuit (Butterworth-Van Dyke) of a QCM operating at the first modal resonance, $f_{s1}$, is shown [1]. 

The equivalent circuit is the parallel of the purely electrical branch (*C*_0_) and of the electromechanical branch, which is a series resonant circuit, representing with inductances the inertial properties of the mechanical system, with capacitance its elastic properties and, with resistances, viscous losses.

Each component of the mechanical system (quartz, additional layer and nonideal liquid) is represented in the electrical domain by the appropriate equivalent components.

In the circuit shown in Figure 1, the functionalization layer is modeled as an additional rigid layer (mass load), but if needed, the model can be refined simply accounting also for its viscoelastic behavior, introducing a further resistor describing the viscous losses. The Newtonian fluid is represented by an inertial load and by a dissipative element.

More in detail, considering an AT-cut quartz with electric permittivity $\epsilon_{Q}^{}=\epsilon_{22}$, shear modulus $\mu_{Q}=c_{66},$ density $\rho_{Q}$, piezoelectric coefficient $d_{26}$, electrode area $A_{e}$, viscosity coefficient $\eta_{Q}$ and mass *m_Q_*, the quartz is represented by the series of $C_{Q1}=8\frac{d_{26}^{2}\mu_{Q}A_{e}}{\pi^{2}t}$, $L_{Q1}=\frac{\rho_{Q}t^{3}\;}{8\mu_{Q}^{2}A_{e}^{}d_{26}^{2}}$ and $R_{Q1}=\frac{\eta_{Q}}{\mu_{q}\;C_{Q1}}$ and by the electrical branch consisting only of the electrical capacitance, which, in air, is $C_{0}=\frac{\epsilon_{Q}A_{e}}{t}$. *C*_0_ can take a slightly different value in-liquid, due to a fringe electric field in the surrounding medium. 

The presence of the additional layer (functionalization and adsorbed target species) with mass Δ*m* is modeled while adding in the series an inductance $L_{Mass}=\frac{\Delta m\;t^{2}}{8\mu_{Q}^{2}A_{e}^{2}d_{26}^{2}}$. 

Finally, the effect of the Newtonian fluid with a density $\rho_{L}$ and viscous coefficient $\eta_{L}$ is represented by a series of a resistor and a nonideal inductors with a resulting impedance [24]:

(3) $$Z_{L}=t^{2}\frac{1+j}{\mu_{q}^{2}A_{e}d_{26}^{2}}\;\sqrt{\pi f\;\rho_{L}\eta_{L}}$$

Usually, since the quartz operates in a small frequency range close to *f_s_*_1_, in Equation (3), it can be assumed that *f* = *f_s_*_1_.

Therefore, we get:

(4) $$L_{L}=\frac{t^{2}}{\mu_{q}^{2}A_{e}d_{26}^{2}}\;\sqrt{\frac{\rho_{L}\eta_{L}}{4\pi f_{s_{1}}}}\;\mathrm{and}\;R_{L}=2\;\pi f_{s_{1}}L_{L}$$

Defining $L_{m},\;C_{m}\;\mathrm{and}\;R_{m},$ as shown in Figure 1, the impedance of the circuit shown in Figure 1 presents a series resonance $f_{s}=\frac{1}{2\pi \sqrt{L_{m}C_{m}}}$ corresponding to the first mechanical modal frequency *f_s_*_1_ and a parallel one $f_{sp}=\frac{1}{2\pi }\frac{\sqrt{C_{m}+C_{0}}}{\sqrt{L_{m}(C_{m}C_{0})}}.$

The two resonances are very close, because *C*_0_ is always much larger than $C_{m}$. 

It can be seen that the series resonance depends only on the mechanical property of the quartz, whereas the parallel resonance depends also on *C*_0_; therefore, it is influenced by the parasitic components due to, for instance, the read-out circuit.

In detail, the series frequency changes due to the presence of an additional mass (and this is the working principle of a microbalance), since the inductance *L_Mass_* contributes to the overall inductance, but, even in the absence of an added mass, the presence of a Newtonian liquid causes a modal frequency shift, due to $L_{L}$.

The shift in this case can be described by the equation derived by Kanazawa and Gordon [25]:

(5) $$\Delta f_{s}\approx -\frac{f_{s}}{2}\frac{\;L_{L}}{L_{Q1}}\approx -f_{s}^{\frac{3}{2}}\sqrt{\frac{\eta_{L}\rho_{L}}{\pi \rho_{Q}\mu_{Q}}}$$

However, only under the assumption of $\frac{\;L_{L}}{L_{Q1}}\ll 1$. Note that this equation holds only for very small changes of $L_{m}$, which is not the case when passing from air to water.

It is well-known that, when placed in an oscillator circuit, the quartz vibrates at a frequency comprised between the series and the parallel resonance frequencies, and the actual oscillation frequency within this range depends on the circuit topology and components.

From the above discussion, it can be concluded that the mechanical properties of the resonator are related by known relationships to the series resonance frequency. Therefore, to obtain information about the mechanical properties either of the fluid or of the added mass exploiting a QCM-based oscillator, the oscillator should operate at a frequency as close as possible to the series resonance of the quartz in any conditions; otherwise, the observed frequency shifts cannot be easily interpreted and related to the variations of the mechanical quantities of interest. 

**Figure 2:**
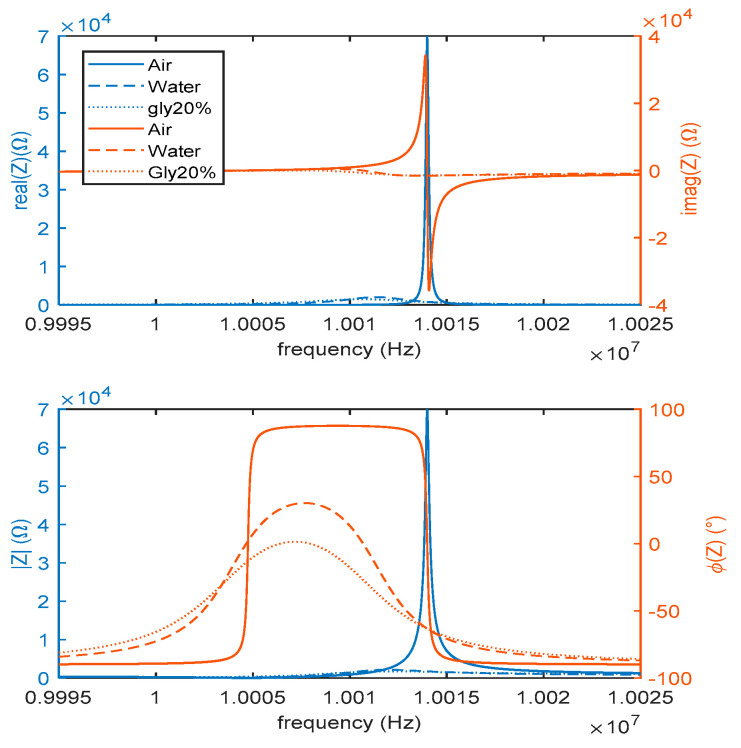
Impedance (real and imaginary parts in the upper plots and magnitudes and phases in the lower plots) of an AT-cut 10-MHz quartz in air (solid lines), in water (dashed lines) and in a solution of water and 20% glycerol (dotted lines). The circuit parameters used to obtain the plots are derived from the experimental data.

To clarify the above discussion, the different impedances of the same 10-MHz AT-cut quartz in air, water and in a solution of water and 20% glycerol are shown in Figure 2. The plots are obtained using values for the different components of the equivalent circuit in Figure 1 assessed either from the theoretical model presented in this section or using experimental data [17]. As expected, beyond shifting the series frequency about 2 kHz, the effect of pure water is to dramatically reduce the Q-factor due to the dissipative effect represented by $R_{L}$, which takes quite a large value (more than ten times the resistance in air). This reduction leads to a less stable behavior and opens many problems in the design of oscillators. In this respect, it must be remembered that these kinds of quartzes are used to measure very small masses. The typical needed mass resolution is in the order of a few nanograms; therefore, the frequency relative resolution has to be smaller than 100 ppb.

**Figure 3:**
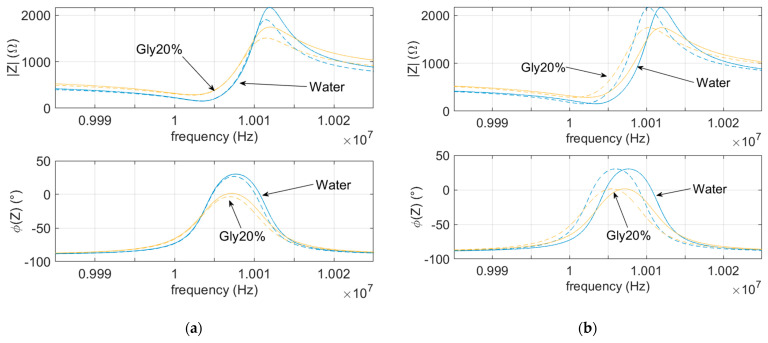
Impedance (magnitude upper plot and phase lower plot) of an AT-cut 10 MHz quartz in water and in a solution of water and 20% glycerol (**a**) with (dashed lines) and without (solid lines) a parasitic capacitance and (**b**) with (dashed lines) or without (solid lines) a 10 μg rigid mass load.

Figure 3a shows the impedances of the 10-MHz quartz in water and in a 20% glycerol–water solution, with and without an additional parasitic capacitance of 2 pF in parallel with *C*_0_, whereas Figure 3b illustrates the effects of an added mass of 10 μg. It can be seen that parasitic capacitances do not affect the series resonance position.

## 3. Oscillator Topology and Working Principle

In QCM-based oscillators for in-liquid applications, the design should, usually, satisfy the following requirements:

- The oscillator should oscillate at a frequency providing an estimation of the series resonance of the quartz.
- One electrode of the quartz should be grounded in order to minimize the parasitic effects due to the fluid in contact with the QCM surface and to simplify the development of the measurement chamber.
- Due to the large reduction of the Q-factor of the quartz, the oscillator circuit must be characterized by a high stability.

**Figure 4:**
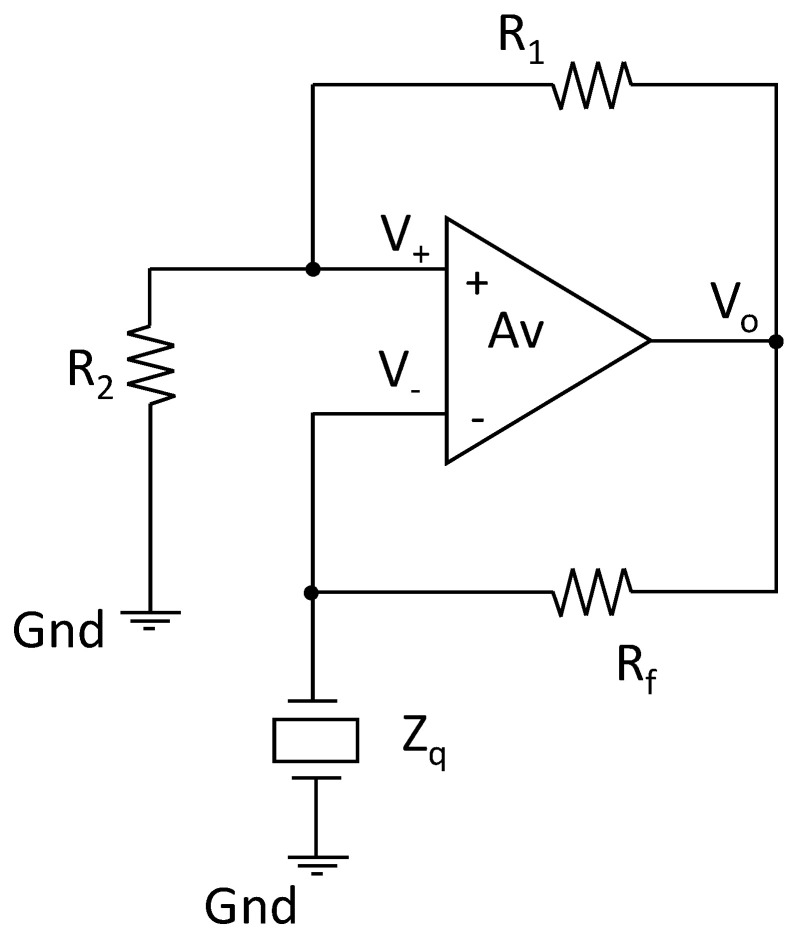
Meacham oscillator topology.

For these reasons, we selected as the oscillator topology the Meacham bridge, shown in Figure 4, which is characterized by a high stability [26], allows for the use of a grounded quartz and oscillates at a zero-phase frequency, $f_{0{}^{\circ}}$. The oscillation frequency can be used as an estimation of the series resonance of the quartz, as required, even if, with the $R_{m}$ different from 0 Ω, the series resonance frequency is close to but does not coincide with this frequency.

In this configuration, we have a loop gain:

(6) $$\beta A_{v}=\left(\beta_{n}-\beta_{p}\right)A_{v}=A_{v}\left(\frac{Z_{Q}}{Z_{Q}+R_{f}}-\;\frac{R_{2}}{R_{2}+R_{1}}\right)$$

where $Z_{Q}$ is the equivalent impedance of the quartz (impedance of the network shown in Figure 1); $A_{v}$ is the open loop gain of the differential amplifier and β, $\beta_{p}$ and $\beta_{n}$ are the overall, the positive and the negative feedback networks gains, respectively. The oscillation conditions are obviously those that grant the existence of a couple of purely imaginary poles of the closed loop gain, i.e., the Barkhausen conditions, requiring the existence of a frequency where the loop gain has a unitary module and a phase equal to −180°.

Ideally, the open loop gain is assumed to be a real number not contributing to the phase of the loop gain. In this case, the oscillator operates at the frequency, $f_{o}$, where the impedance of the quartz is purely real; therefore, at the oscillation frequency, it can be written:

(7) $$\beta_{n}\left(f_{o}\right)=\frac{Z_{Q}\left(f_{o}\right)}{R_{f}+Z_{Q}\left(f_{o}\right)}$$

where $Z_{Q}\left(f_{o}\right)\in ℜ$. Note that, to satisfy the phase Barkhausen condition, it must be granted that $\beta_{p}\left(f_{o}\right)>\beta_{n}\left(f_{o}\right)$; therefore, in any working condition, the following inequality should be satisfied:

(8) $$\frac{R_{1}}{R_{2}}<\frac{R_{f}}{Z_{Q}\left(f_{o}\right)}$$

Therefore, in the Meacham oscillator, at the oscillation frequency, the quartz operates as a pure resistive component; therefore, $f_{o}=f_{0{}^{\circ}}$, and, if $R_{m}$ is sufficiently small, we have $Z_{Q}\left(f_{o}\right)\approx R_{m}$ and $f_{o}=f_{0{}^{\circ}\;}\approx f_{s}$.

The inequality in (8) is particularly critical for QCMs operating in-liquid. In fact, these devices present a largely variable value of $R_{m}$ due to the contributions of the surrounding liquids, which can have different viscosities. 

Besides the phase condition (Equation (7)), the marginal stability of the system is granted by selecting the value of $A_{v}$ to also satisfy the Barkhausen module condition, which is:

(9) $$A_{v}\left(\;\frac{R_{2}}{R_{2}+R_{1}}-\;\frac{Z_{Q}\left(f_{o}\right)}{Z_{Q}\left(f_{o}\right)+R_{f}}\right)=1$$

Note that, if Equation (9) is satisfied, given the value of $A_{v}$ and those of the resistances *R*_1_, *R*_2_ and *R_f_*, it is possible to derive the value of $Z_{Q}\left(f_{o}\right)\approx R_{m}$.

Unfortunately, this equality cannot be perfectly met in the reality, and for a QCM, this problem is particularly critical, because the value of $Z_{Q}\left(f_{o}\right)$ varies during operations. 

In any case, the loop gain module should be as close to 1 as possible but larger than 1; otherwise, the oscillator behaves as a stable system, and its output is not persistent.

These considerations clarify how real oscillator circuits never behave as pure marginally stable systems; on the contrary, they can be modeled in the linear regime with poles with the real part larger than zero, and they always also operate in nonlinear regions. As a consequence, the imaginary part of the unstable poles, which sets the frequency of the oscillator output, does not coincide with the frequency of the marginally stable poles. 

To deepen this discussion, the overall gain of the amplifier with feedback, $A_{f}$, can be written in the Laplace domain in the following way:

(10) $$A_{f}\left(s\right)=\frac{A_{v}\left(s\right)}{1+\beta \left(s\right)A_{v}\left(s\right)}=\frac{N\left(s\right)}{P\left(s\right)\left(\frac{s^{2}}{\omega_{N}^{2}}-\frac{2\zeta }{\omega_{N}}s+1\right)}$$

where N(s) and $P\left(s\right)\left(\frac{s^{2}}{\omega_{N}^{2}}-\frac{2\zeta }{\omega_{N}}s+1\right)$ are polynomials in *s*.

By describing the gain of the amplifier in Equation (10), the denominator factor, responsible for the oscillating behavior, is written in the Bode form. Therefore, the couple of unstable poles responsible for the oscillation are:

(11) $$p_{1,2}=\zeta \omega_{N}\pm j\omega_{N}\sqrt{1-\zeta^{2}}$$

where *ζ* and $\omega_{N}$ are positive real numbers, and $\alpha =\zeta \omega_{N}$ is the positive real part of the poles. Consequently, the response to the initial condition of the system contains a nonvanishing contribution due to this couple of poles that can be written as:

(12) $$V_{0}{}_{unst}\left(t\right)=Ae^{\zeta \omega t}\cos\left(\omega_{N}\sqrt{1-\zeta^{2}}t+\phi \right)$$

where *A* and ϕ are the parameters related to the initial conditions. Therefore, the oscillator operates at the frequency $f_{o},$ defined as follows:

(13) $$f_{o}=\frac{\omega_{N}}{2\;\pi }\sqrt{1-\zeta^{2}}$$

If Equation (9) is perfectly satisfied, and if $R_{m}\;$ is sufficiently small, then the poles are marginally stable, and *ζ* is equal to zero; therefore, we can write:

(14) $$\omega_{N}=2\pi f_{S}$$

In this case, the oscillator frequency estimates the series resonance frequency as desired.

On the other hand, if the loop gain module at the frequency selected by the phase condition is larger than one, then the frequency of the oscillator is different from the searched resonance frequency, and we can approximate the difference,$\;e_{f}$, between the two frequencies as follows:

(15) $$e_{f}=f_{s}-f_{o}\approx f_{s}\left(1-\sqrt{1-\zeta^{2}}\right)$$

It is interesting relating the deviation $e_{f}$, which is a measurement error, to the magnitude of the loop gain module. To this end, we propose a simplified analysis considering $A_{v}$ as a real number and the value of $R_{m}$ small. To obtain the relationship between the oscillator characteristics, i.e., how much the loop gain is larger than 1 and the measurement error, it is convenient to write the denominator of the amplifier gain, $A_{f}$, defined in Equation (10), with the following equation:

(16) $$P\left(s\right)\left(\frac{s^{2}}{\omega_{N}^{2}}-\frac{2\zeta }{\omega_{N}}s+1\right)=D_{\beta }\left(s\right)+N_{\beta }\left(s\right)A_{v}=\left(1+A_{v}\beta \left(s\right)\right)D_{\beta }\left(s\right)$$

where $N_{\beta }\left(s\right)$ and $D_{\beta }\left(s\right)$ are the numerator and the denominator of β, respectively. At the frequency satisfying the phase condition, which is approximately *f_s_*, *β* is a real number; therefore, $D_{\beta }\left(f_{o}\right)+N_{\beta }\left(f_{o}\right)A_{v}$ is either a real number and its imaginary part is zero and or a pure imaginary number with a null real part.

(17) $$\alpha \left(s\right)=\frac{s^{2}}{\omega_{N}^{2}}-\frac{2\zeta }{\omega_{N}}s+1\;\mathrm{and}\;\Gamma =\left|1+A_{v}\beta \left(f_{o}\right)\right|\in ℜ.$$

Hence, in the first case, $N_{\beta }\left(f_{o}\right)$ and $D_{\beta }\left(f_{o}\right)\in ℜ,$ we have:

(18) $$\left|Re\left(P\left(f_{o}\right)\right)Re\;\left(\alpha \left(f_{o}\right)\right)-Im\left(P\left(f_{o}\right)\right)Im\left(\alpha \left(f_{o}\right)\right)\right|=\left|1+\beta \left(f_{o}\right)A_{v}\right|\;\left|D_{\beta }\left(f_{o}\right)\right|$$

While, in the second case, $N_{\beta }\left(f_{o}\right)$ and $D_{\beta }\left(f_{o}\right)\in ℑ$, we have:

(19) $$\left|Re\left(P\left(f_{o}\right)\right)Im\;\left(\alpha \left(f_{o}\right)\right)+Im\left(P\left(f_{o}\right)\right)Re\left(\alpha \left(f_{o}\right)\right)\right|=\left|1+\beta \left(f_{o}\right)A_{v}\right|\;\left|D_{\beta }\left(f_{o}\right)\right|$$

At the frequency *f_s_,* we have $Re\left(\alpha \left(f_{s}\right)\right)$ = 0 and $Im\left(\alpha \left(f_{s}\right)\right)=2\zeta$.

So, in the first case, we derive:

(20) $$2\zeta \approx \Gamma \frac{\left(R_{m}+R_{f}\right)\left(R_{2}+R_{1}\right)}{\left|Im\left(P\left(f_{o}\right)\right)\right|}=\frac{\left(R_{m}R_{2}+R_{1}R_{m}+R_{f}R_{2}+R_{f}R_{1}\right)+A_{v}R_{m}R_{1}-A_{v}R_{f}R_{2}}{Im\left(P\left(f_{o}\right)\right)}$$

While, in the second case:

(21) $$2\zeta \approx \Gamma \frac{\left(R_{m}+R_{f}\right)\left(R_{2}+R_{1}\right)}{Re\left(P\left(f_{o}\right)\right)}$$

The last equations describe accurately the behavior of the oscillator for a small value of $R_{m}$, whereas, for larger values of $R_{m},$ the oscillator frequency deviates from $f_{o}$ in a different manner.

In any case, Equations (20) and (21) show that the larger the loop gain is with respect to 1, the larger the coefficient ζ and the deviation between the oscillator output frequency and the quartz series resonance frequency. Moreover, and more importantly, this error is not independent from the measurement conditions, and it cannot be compensated, since it varies with $R_{m}$. Concluding the deviation of the oscillator frequency from the measurand is a function of $A_{v}$ and of the characteristics of the fluid.

This discussion clarifies that the operating frequency of the oscillator deviates from the desired one (in this case, the series resonance of the quartz) if the loop gain module is much larger than 1 and that the deviation depends on the working conditions of the QCM.

To mitigate these problems, we propose a Meacham oscillator for AT-cut 10-MHz quartzes based on a large band differential amplifier with variable gain (VGA) VCA842, by Texas Instruments (Dallas, TX, USA). With this solution, it is possible to adaptively change the open loop differential gain, $A_{v},$ in Equation (9) to maintain the loop gain as close to 1 as possible, irrespective of the working conditions of the quartz.

### 3.1. Effect of the Phase of the Amplifier

The oscillator working frequency corresponds exactly to the zero-phase frequency of the quartz only in the case of an ideal amplifier; in reality, due to the behavior of a real amplifier, the measured frequency deviates both from the series resonance frequency and from the zero-phase frequency, and the effect of the amplifier nonidealities grows, increasing the acoustic losses. 

In particular, the assumption of $A_{v}$ having a 0° phase around 10 MHz (typical working frequency for QCM) accepted until now is critical; this means that the amplifier has to be chosen with extreme care, because the phase lag of the amplifier is compensated by the phase of the feedback network gain, bringing the quartz to operate far from the series resonance. 

The selected amplifier phase is smaller than 6° at 10 MHz for all the possible gain values, granting satisfactory operations. In this subsection, the effect of the amplifier phase lag is evaluated.

**Figure 5:**
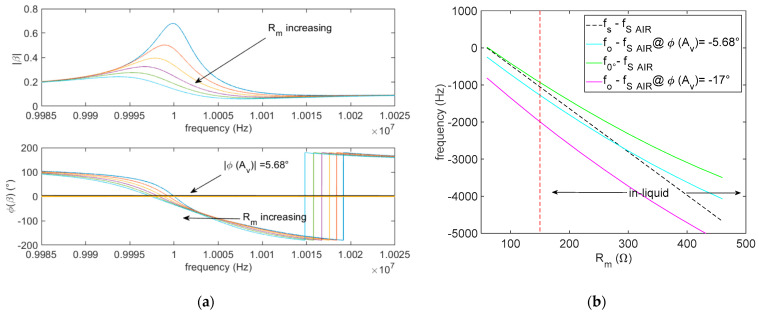
(**a**) *β* as a function of the frequency at different values of $R_{m}$: upper plot *β* module and lower plot phase of *β*. (**b**) Comparison between the series resonance of an AT-cut 10MHz quartz operating in a Newtonian fluid as a function of the series resistance, $f_{0{}^{\circ}}$ (which would be the oscillator frequency in the case of an ideal amplifier) and $f_{o}$, which is the oscillator frequency accounting for the real amplifier behavior, considering *R*_2_/*R*_1_ = 10 and $R_{f}$ = 200 Ω.

In Figure 5, the oscillator is considered to operate at the optimum gain magnitude so that Equation (9) is satisfied, replacing *A_v_* with |*A_v_(f*_*o*_*)*|. In this figure, the oscillator working frequency (cyan line) is compared to the quartz series resonance frequency (black dashed line) and to the zero-phase frequency (green line) in the case of *R*_2_*/R*_1_ = 10 and *R_f_* = 200 Ω and taking into a dominant pole the transfer function for the amplifier gain. The comparison points out that the deviation is acceptable also when the fluid is characterized by large losses. In fact, it can be seen that the effect introduced by the amplifier phase lag (cyan curve in Figure 5) is the one providing an overestimation of $f_{0{}^{\circ}}$, but since $f_{0{}^{\circ}}$ itself is an underestimation of $f_{s}$, the small amplifier phase lag partially compensates the error induced by the large acoustic losses in the liquid and provides a working frequency of the oscillator closer to the series resonance frequency. Obviously, if the phase lag becomes large, the error becomes very high, as can be seen in Figure 5, where the magenta line shows the effect of a large amplifier phase lag.

**Figure 6:**
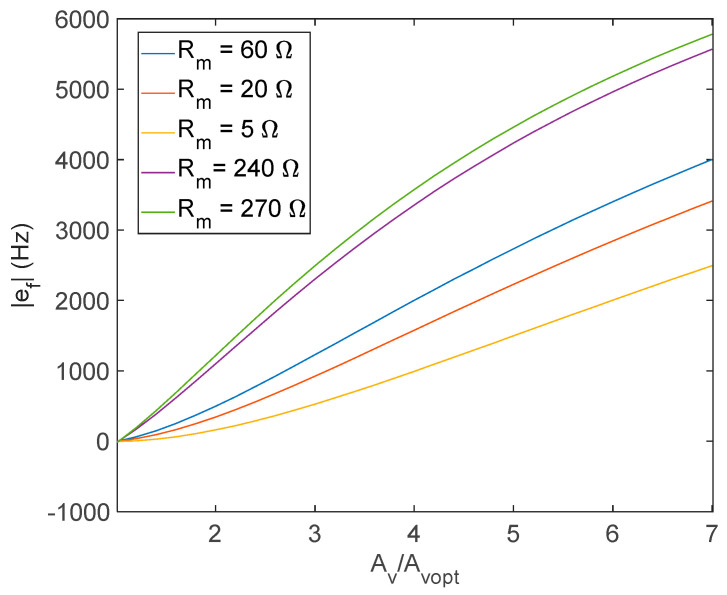
The error *e_f_* defined in Equation (15) evaluated for an AT-cut 10 MHz quartz, considering *R*_2_*/R*_1_ = 10 and *R_f_* = 200 Ω, and a phase lag of the amplifier of −5.68°.

Nonetheless, the effect of even a small phase lag becomes very important when operating with a gain that does not satisfy Equation (9). This can be seen extending through simulations, the results obtained from the theoretical analysis presented in the previous subsection concerning the frequency error *e_f_* due to the operation with large gains of the amplifier, taking into account also the frequency behavior of the amplifier. As an example, Figure 6 shows the frequency error defined in Equation (15) for an AT-cut 10-MHz quartz, considering the circuit components reported in the legend, as a function the amplifier open loop gain (direct current (DC)value) normalized with respect to the value $A_{vopt}$ (DC value), which is the value of the gain needed to satisfy Equation (9). In this case, even a small phase lag of −5.68° introduced by the selected amplifier causes very large errors.

### 3.2. Evaluation of the Series Resistance 

**Figure 7:**
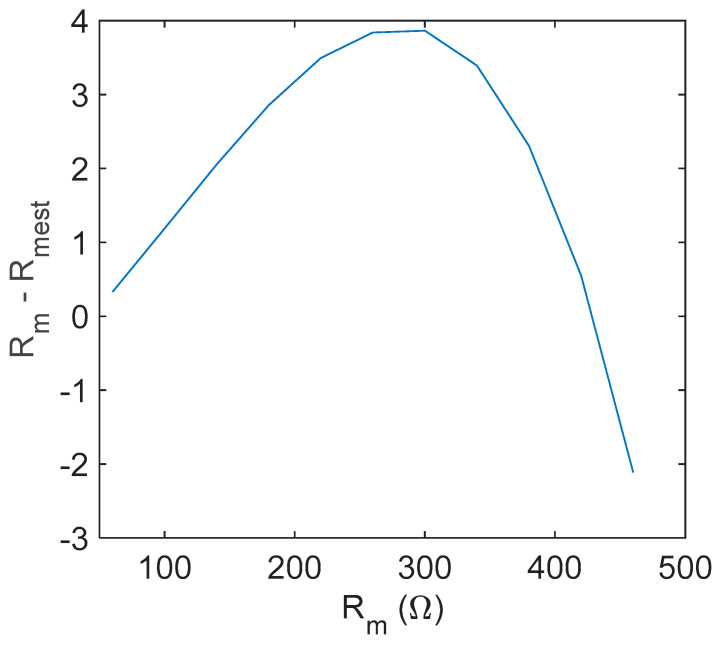
Error in the estimation of $R_{m}$ from Equation (22) for an AT-cut 10 MHz quartz considering *R*_2_/*R*_1_ = 10 and $R_{f}$ = 200 Ω.

If the open loop amplifier gain $A_{v}\;=\;A_{vopt}$, which is the value that verifies Equation (9), is known, then the value of $R_{m}$ can be evaluated by inverting the same equation, under the assumption of small losses and ideal amplifier behavior, obtaining:

(22) $$R_{m}=\frac{\frac{R_{2}R_{f}}{R_{1}+R_{2}}-\frac{1}{A_{v_{opt}}}R_{f}}{1+\frac{1}{A_{v_{opt}}}-\frac{R_{2}}{R_{2}+R_{1}}}$$

where, due to the low loss assumption, we consider $Z_{Q}\left(f_{o}\right)=R_{m}$. Indeed, as diffusely discussed in the previous subsections, the assumptions used to derive Equation (22) are not fully satisfied, and Equation (22) provides only an estimation of $R_{m}$ with a certain degree of uncertainty. To evaluate the quality of this estimation, simulations were performed, taking into account the behavior of the real amplifier in terms of the frequency response and considering also large values of $R_{m}$. The simulation results showed that the accuracy of the $R_{m}$ estimation based on Equation (22) is really very high (the error is always smaller than few Ωs). As an example, Figure 7 shows the error between the “true” value of $R_{m}$ used for the simulations of the quartz behavior and the value obtained from Equation (22), considering also the amplifier nonideality (as in Figure 6), i.e., accounting for the amplifier phase lag.

Finally, concerning the uncertainty of the estimation related to the uncertainty of the gain estimation, we can write:

(23) $$u\left(R_{m}\right)\approx \frac{\partial R_{m}}{\partial A_{v}}\;u\left(A_{v}\right)=\frac{R_{f}}{A_{v}^{2}{\left(1+\frac{1}{A_{v}}-\frac{R_{2}}{R_{1}+R_{2}}\right)}^{2}}\;u\left(A_{v}\right)$$

which, considering operating close to the optimum gain, can be expressed as a function of *R_m_* as follows:

(24) $$u\left(R_{m}\right)=\frac{{\left(R_{m}R_{1}-R_{f}R_{2}\right)}^{2}}{R_{f}{\left(R_{1}+R_{2}\right)}^{2}}\;u\left(A_{v}\right)$$

Showing that there exists an optimum value of *R_m_*,$\;R_{m}^{*}=\frac{R_{f}R_{2}}{R_{1}}$, where the estimation of *R_m_* is robust with respect to the estimation errors of the optimum gain.

As a concluding remark, it is shown that the estimation of the series resistance value is found to be significantly more robust than the one of the series resonance frequency, and as such, $R_{m}$ is a very useful parameter, which should be taken into account for the correct interpretation of measurements.

## 4. Measurement System Architecture and Gain Adjustment Procedure

**Figure 8:**
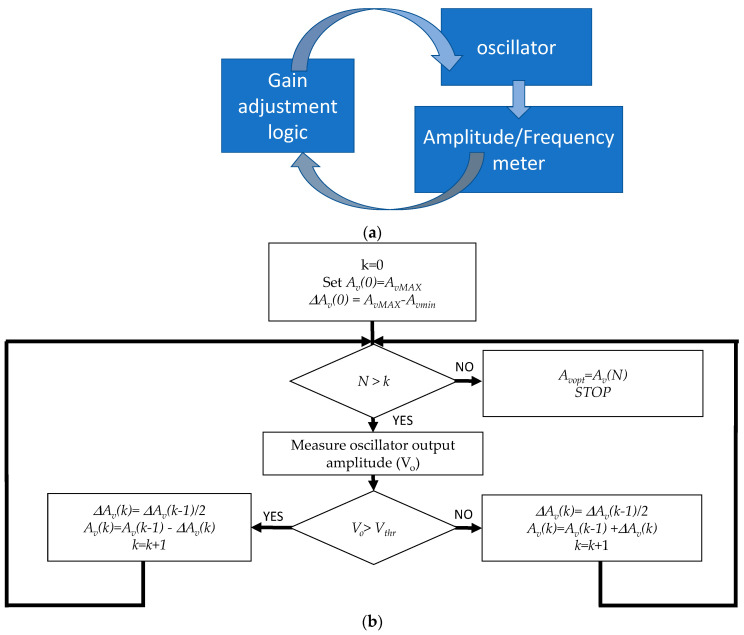
(**a**) Schematics of the building blocks operations of the proposed system. (**b**) Gain adjustment strategy.

The proposed measurement system is composed by the oscillator, an amplitude meter able to monitor the oscillator output amplitude and a control block that provides gain adjustment with a feedback loop exploiting the measured oscillator output amplitude, as shown in Figure 8.

Gain adjustment is provided by a digital controller (the block named “gain adjustment logic” in Figure 8) that executes the iterative dichotomic search of the optimum gain value illustrated in Figure 8b with the objective of minimizing the quantity $\gamma =\left|A_{v}\left(\;\frac{R_{2}}{R_{2}+R_{1}}-\;\frac{Re\left(Z_{Q}\left(f_{o}\right)\right)}{Re(Z_{Q}\left(f_{o}\right))+R_{f}}\right)\right|-1$ in N steps.

Each step lasts a time *T_s_*, so the search process concluded in *N* iterations, a measurement sample of the oscillator frequency, $f_{o}$, and $R_{m}$ is provided after a time equal to *t_meas_* = *NT_s._* For the real-time monitoring of adsorption phenomena, *t_meas_* must be less than about 2 s.

The search algorithm is based on the two following ideas:

- the system works in a predefined range of $R_{m}$, with $R_{m}$ < $R_{mMAX}$.
- At the start, the gain is set at the maximum value, and this is sufficient for the oscillator to also work in the case of $R_{m}$ = $R_{mMAX}$ (minimum Q-factor), with the oscillator behaving as an unstable (or marginally stable) system with $A_{v}$ ≥ $A_{vopt}$.
- At each search process step, the amplitude of the oscillator output signal is measured and used to detect the presence of persisting oscillations.

The search procedure, described in Figure 8b, is based on reducing the gain on a dichotomic basis, checking for the presence of oscillations with an amplitude larger than a predefined threshold related to the noise floor and taking as $f_{o}$ the frequency measured at the last step. The corresponding gain is the estimated $A_{vopt}$ and can be used to assess the value of $R_{m}$ based on Equation (9).

Using *N* search steps, the gain can be adjusted with a resolution given by $\Delta A_{v}=\frac{A_{v}{}_{MAX}-A_{v}{}_{MIN}}{2^{N}}\;,$ and consequently, using the same procedure adopted to derive the uncertainty as in Equation (24), the resolution of the $R_{m}$ measurements is $\Delta R_{m}=\frac{\partial R_{m}}{\partial A_{v}}\frac{A_{v}{}_{MAX}-A_{v}{}_{MIN}}{2^{N}}$.

Notice that, as usual, accurate measurements need longer measurement times; therefore, the settings in terms of gain resolution and measurement time must be selected as a trade-off.

### Measurement System Setup

**Figure 9:**
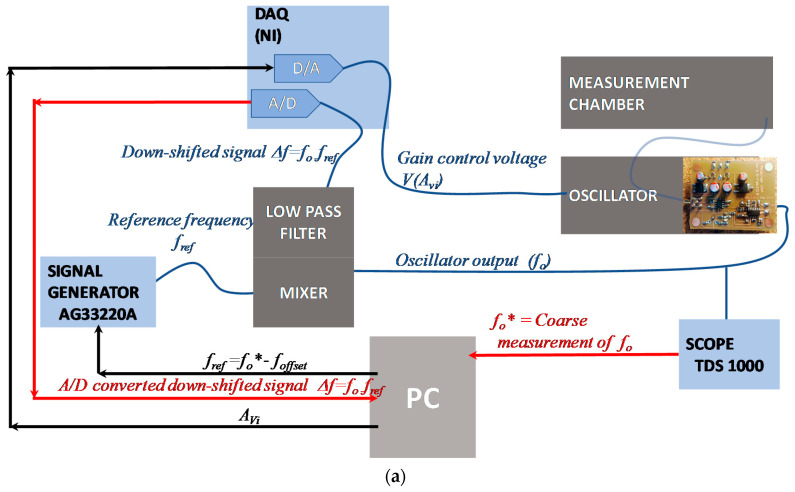

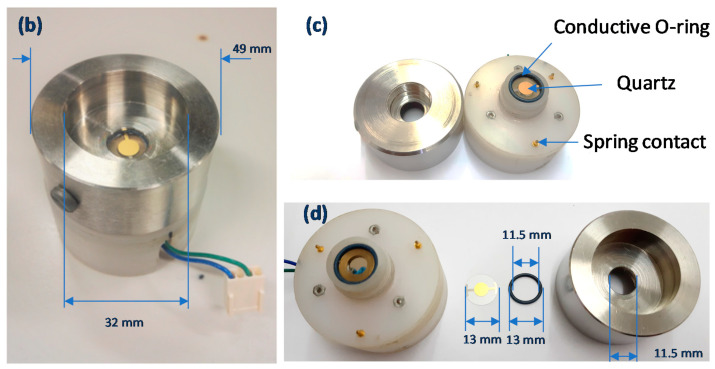
(**a**) Laboratory set-up for the characterization of the proposed system. (**b**–**d**) Measurement chamber: assembled (**b**) and building blocks views (**c**,**d**).

The system, described in principle in Figure 8, was implemented in the laboratory to test the performance of the proposed oscillator. The implemented measurement set-up is described in Figure 9. In particular, the AT-Cut 10-MHz quartz (gold electrodes having a diameter of 0.6 cm) was placed in an ad-hoc-built measurement chamber that allowed for static measurements in liquid. The measurement chamber, shown in Figure 9b–d, has a cylindrical shape with a 49-mm outer diameter and 50-mm height. The chamber is composed of two main blocks (see Figure 9c,d), the bottom one is a polyvinyl chloride (PVC) material and hosts a proper designed holder and connector for the quartz. The quartz is sandwiched between two conductive O-rings. The top block (cap) is stainless steel and presents a central hole, under which the quartz is placed. The steel cap is grounded by means of spring-loaded contacts and connected to the upper quartz electrode. The quartz upper surface acts as the bottom wall of a measurement well with a diameter of 11.5 mm and a height of 2 mm, guaranteeing the liquid deposition on the quartz surface. All the tests in liquid are performed using the volume of the liquid such that the thickness of the liquid layer is much larger than the extinction depth $\delta^{\prime }=\sqrt{\frac{2\eta_{L}}{\omega_{s}\rho_{L}}}$ [27]; therefore, the liquid can be seen as a semi-infinite layer.

The quartz is connected to the developed Meacham oscillator; the gain of the VGA is set through the 16-bit D/A converter of an acquisition board (NI PCI 6052E, D/A) and a conditioning amplifier.

With reference to Figure 4, the Meacham bridge was realized using *R*_1_ = 2.4 kΩ, *R*_2_ = 24 kΩ and *R_f_* = 200 Ω (which are the same values considered for the all the simulations whose results were presented in the previous sections).

The amplitude and the frequency of the oscillator output are digitally measured by a LabView VI executed by a PC after having acquired, with the same acquisition board (16-bit A/D), a frequency downshifted version of the signal. The frequency downshift of the oscillator output is obtained using an ad-hoc developed circuit hosting a mixer and a low-pass filter used to mix the oscillator output with a reference signal the with frequency $f_{ref}$ generated by a signal generator AG33220A (Agilent, Santa Clara, CA, USA). The $f_{ref}$ is automatically selected by exploiting a coarse measurement of $f_{o}$, performed during the set-up phase, with $A_{v}\;$=$\;A_{vMAX}$, such that $f_{ref}\;$=$\;f{\;}_{o}^{*}\;$− $f_{offset}$. The frequency $f_{offset}$ is selected to guarantee, during the whole measurement, $f_{o}\;$> $f_{ref}$ and $\Delta f=\;f_{o}-f_{ref}$ in the kHz range.

The mixer output signal is acquired with a suitable sampling frequency for the time windows of the duration *t_acq_* < *T_s_*. The gain adjustment algorithm (Figure 8b) is implemented by the same VI controlling the acquisition and performing the amplitude measurements.

The same VI assesses also the frequency of the acquired signal at each search process step, using an accurate single tone estimation algorithm.

The selected acquisition time is 150 ms, and the optimum gain search procedure consists of a number of steps, *N*, that can be selected in the range 9–12. The measurement time, *t_meas_*, was fixed at 2 s; therefore, each two seconds, the system provides and saves a measurement of $f_{o}$ performed with the optimum gain (obtained at the last step of the gain adjustment procedure) and, for comparison sake, a measurement of $f_{o}$ obtained at the starting step of the gain adjustment procedure (with maximum gain).

The TI VCA842 differential amplifier has a gain range 1/10 < *A_v_* <10, but, in this application, we consider *A_vmin_* = 1 and a linear analog control of the gain value, obtained by means of a voltage input *V_G_.* The gain is related to the control voltage by the following equation:

$$A_{v}=4.90\;V^{-1}\left(V_{G}+1V\right)$$

The search strategy resolution, which is considered as the largest source of uncertainty for the estimation of *A_vopt_*, is related to the number of steps that are used to adjust the gain. In the presented case, the number of steps, *N*, used can be selected in the range 9–12; we have, therefore:

$$\Delta A_{v}=\frac{A_{v}{}_{MAX}-A_{v}{}_{min}}{2^{N}}<\frac{10}{2^{N}}\;N=9,\;\ldots ,12$$

Exploiting the results of Equation (24), and using the circuit component values, we found that the obtainable resolution for the *R_m_* estimation is in the range 2 Ω–4 Ω with *N* = 9, whereas, with *N* = 12, the resolution is improved a factor of 8 and lies in the range 0.25 Ω–0.5 Ω.

**Figure 10:**
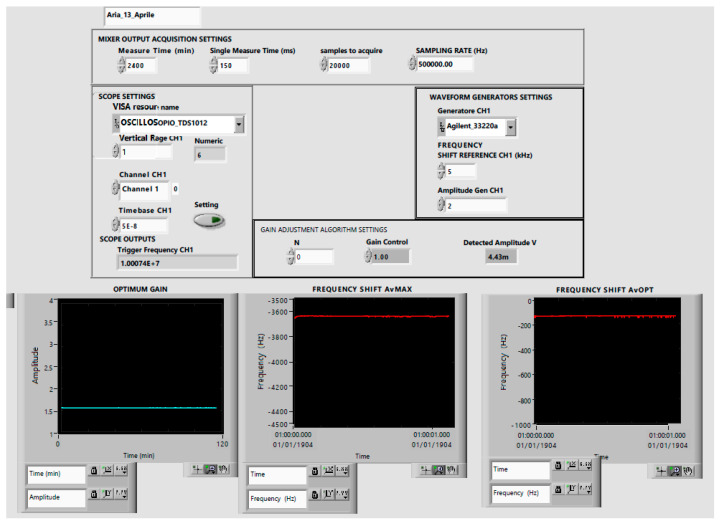
VI front-panel.

The VI front-panel is shown in Figure 10.

## 5. Experimental Results

To test the theory discussed in the previous section, *f_o_* and *R_m_* were monitored in different experimental conditions for time windows 25-min-long performing the optimum gain search (*A_vopt_*); simultaneously, the *f_o_* value obtained without using the gain adjustment procedure at the maximum gain *A_vMAX_* was also acquired. Table 1 shows the mean deviation *e_f_* between the two frequencies ($i.e.,\;e_{f}=f_{o}@A_{vopt}-\;f_{o}@A_{vMAX}$) in the tested different conditions. The experimental measurements were repeated at least five times for each of the considered test cases in the same environmental conditions.

In particular, the reported results concern tests performed in the air, pure water (*η*_H2O_ = 8.4 × 10^−4^ Pa∙s, *ρ*_H2O_ = 1000 kg/m^3^) and a mixture of pure water and 10% of glycerol (*η_gly_* = 1.5 Pa∙s, *ρ_gly_* = 1260 kg/m^3^).

Assuming *f_o_* as a good estimation of *f_s_* in the case of the adjusted gain, we find an error due to the loop gain module inaccuracy of about 3 kHz in the air (*e_f_* = 3377 Hz) and a larger error in the liquid; these results are in accordance with the simulation analysis reported in Figure 6. Moreover, operating at an optimum gain usually increases the stability of the oscillator, especially when the optimum value is far from the maximum gain value.

The standard deviations reported in the table describe the short-time frequency stability provided by the oscillator in different test conditions. Nevertheless, the analysis of repeated measurements showed larger deviations that can be explained by the variations of the influence quantities (e.g., ambient temperature varied approximately in a 5 °C interval during the measurement campaign). The Allan variance was assessed to gain information on the variability/stability of the oscillation frequency over time in different conditions: in air and in water. The standard deviation values of the frequency variations for one hour and one day are: <3 Hz, ≈20 Hz in the air and <20 Hz, ≈50 Hz in the liquid.

**Figure 11:**
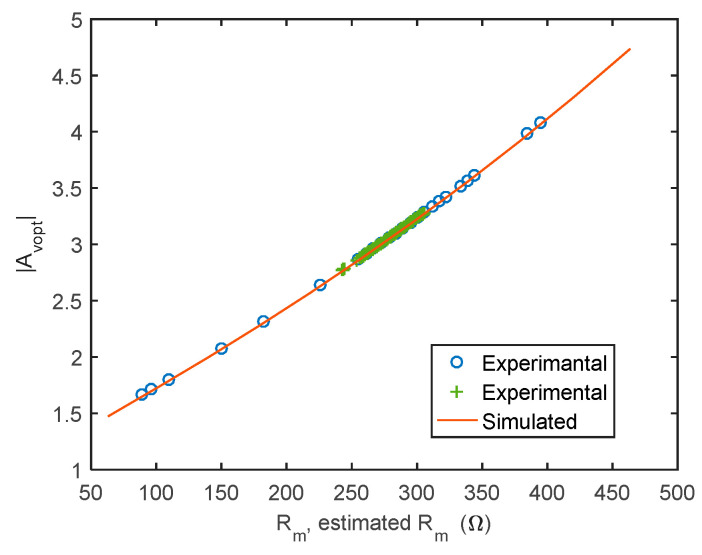
Optimum gain predicted by the simulations (solid line) in the same conditions used to derive Figure 5, Figure 6 and Figure 7 as a function of the “true” series resistance. Optimum gain provided the adjustment procedure experimentally (markers) as a function of the estimated *R_m_*.

Figure 11 reports the optimum gain predicted by simulations (solid line) in the same conditions used to derive Figure 5, Figure 6 and Figure 7 as a function of the series resistance and the optimum gain provided by the adjustment procedure experimentally (markers), showing a very good accordance.

**Figure 12:**
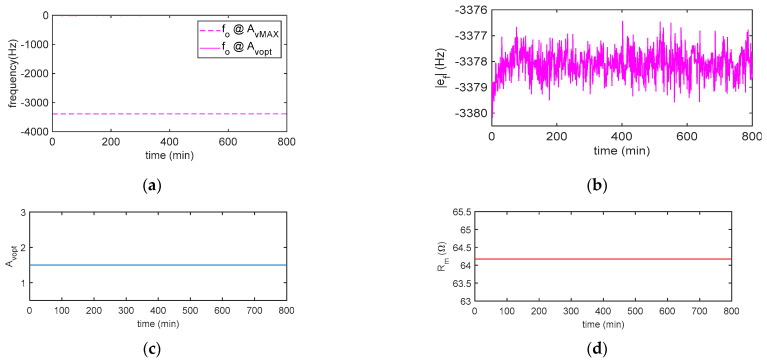
Measurement results obtained in the air in approximately 13 h: (**a**) oscillation frequency *f_o_* at the maximum $\left(A_{vMAX}\right)$ and optimum gain $\left(A_{vOpt}\right)$ and (**b**) frequency deviation $e_{f}=f_{o}@A_{vOpt}-f_{o}@A_{vMAX}$. (**c**) Estimated $A_{vOpt}$ and (**d**) estimated *R_m_*. *N* = 10.

Figure 12 reports the results obtained in a 13-h measurement performed in the air with the quartz placed in the chamber and shows the performance of the system in terms of the short time stability. It is worth noticing that the constraint exerted by the placement of the quartz in a chamber for in-liquid applications causes a perturbation of the resonant system accompanied by a modest reduction of the Q-factor. Therefore, the short-term stability of the measurements in the air results are somewhat worse than the ones obtainable for in-gas applications where the QCM fixing is obtained by suspending the quartz with small springs, which cause a negligible mechanical load.

**Figure 13:**
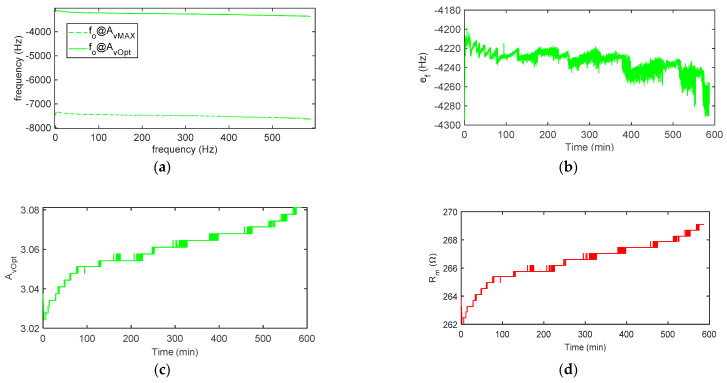
Measurement results obtained in water and 10% glycerol during approximately 10 h: (**a**) oscillation frequency *f_o_* at the maximum $\left(A_{vMAX}\right)$ and optimum gains $\left(A_{vOpt}\right)\;\mathrm{and}\;$(**b**) frequency deviation $e_{f}=f_{o}@A_{vOpt}-f_{o}@A_{vMAX}$. (**c**) Estimated $A_{vOpt}$ and (**d**) estimated *R_m_*. *N* = 12.

Figure 13 reports the results obtained in a 10-h measurement performed in a solution of pure water and 10% of glycerol. The effect of evaporation of the water during the measurement, accompanied by an increase of the solution viscosity, can be seen both from the decreasing of the frequency shift with time and from the increasing of the optimum gain.

**Figure 14:**
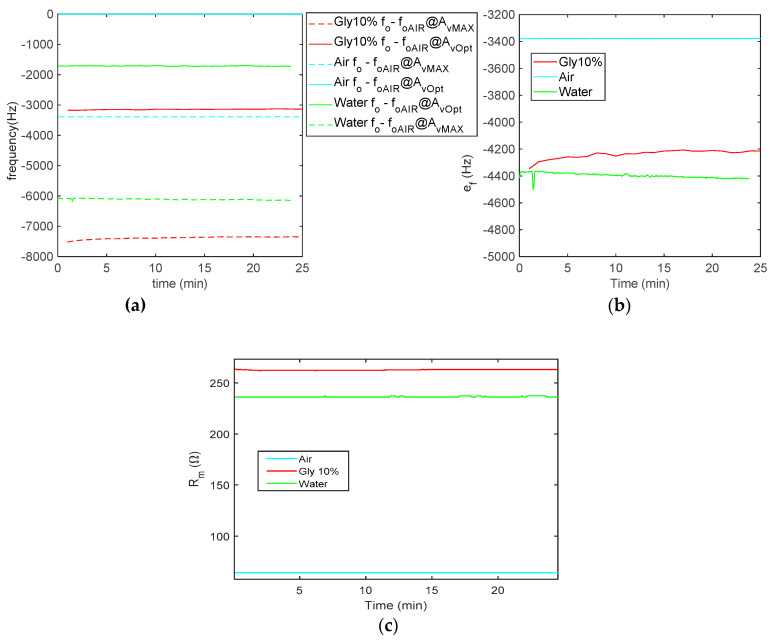
Measurement results obtained in the air (*N* = 12), water (*N* = 10) and 10% glycerol (*N* = 12) during 25 min: (**a**) oscillation frequency shifts, *f_o_* − *f_oAIR_*, where *f_oAIR_* is the oscillator frequency in air and at optimum gain; dashed lines: shifts at maximum $\left(A_{vMAX}\right)$ and continuous lines: shifts at optimum gain $\left(A_{vOpt}\right)$. (**b**) Frequency deviation $e_{f}=f_{o}@A_{vOpt}-f_{o}@A_{vMAX}$. (**c**) Estimated *R_m_*.

The results obtained in the different conditions are compared in Figure 14, showing that the effect of the gain setting is relevant particularly when considering fluids with a large difference in viscosity.

**Figure 15:**
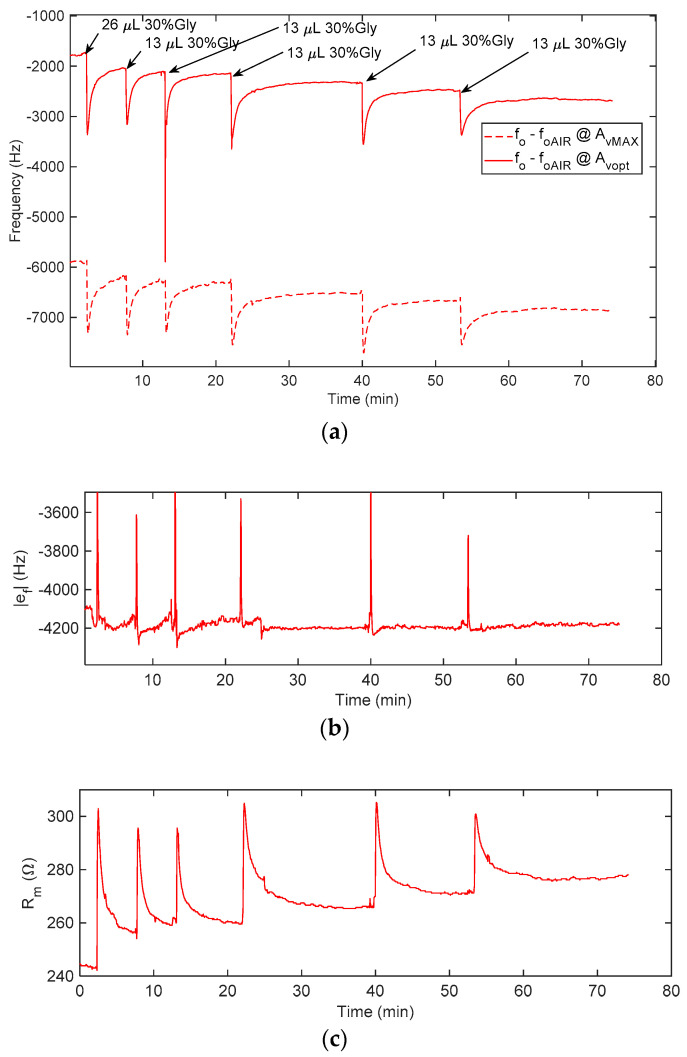
QCM monitoring starting from pure water and adding, with a micropipette, subsequent doses of 13 μL or 26 μL of a solution with 30% glycerol, obtaining solutions with glycerol concentrations of 0%, 4.7%, 6.5%, 8.1%, 9.5%, 10.7% and 11.8%. (**a**) Oscillation frequency shifts, *f_o_* − *f_oAIR_*, where *f_oAIR_* is the oscillator frequency in the air and at the optimum gain; dashed lines: shifts at maximum $\left(A_{vMAX}\right)$ and continuous lines: shifts at the optimum gain $\left(A_{vOpt}\right),$ *N* = 12. (**b**) Frequency deviation $e_{f}=f_{o}@A_{vOpt}-f_{o}@A_{vMAX}$. (**c**) Estimated *R_m_*.

Finally, Figure 15 shows a dynamical test, *f_o_*, was monitored starting from pure water (140 μL) and adding, with a micropipette, subsequent doses of 13 μL or 26 μL of a solution of water with 30% glycerol, obtaining solutions with glycerol concentrations of 0%, 4.7%, 6.5%, 8.1%, 9.5%, 10.7% and 11.8%. The transients induced by the perturbation due to the drop cast can be seen. The glycerol solution is dropped manually with a micropipette, and the added solution has a different density and temperature with respect to the one in the well, so, when the drop falls (even if we tried to perform this operation as smoothly as possible), a fluid dynamical transient is expected before the solution diffuses and reaches a steady state. Due to the manual deposition, the transients obtained with the individual drop depositions are somewhat different.

These measurements show that the gain successfully adapts itself to the different measurement conditions, compensating as needed for the change of *R_m_* during measurements.

**Figure 16:**
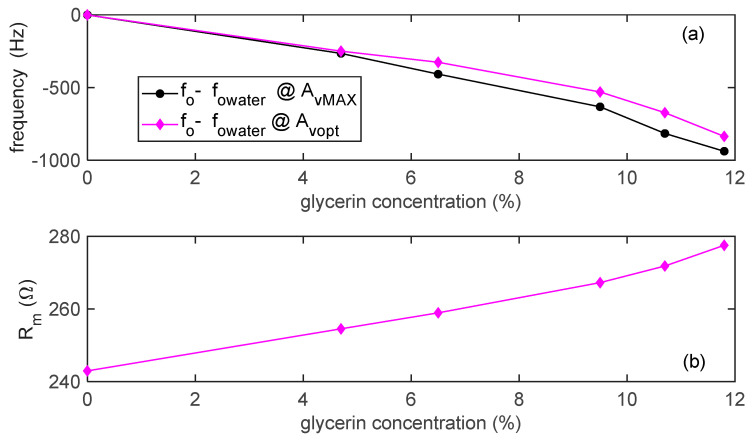
(**a**) Oscillation frequency shifts, *f_o_* − *f_owater_* (where *f_owater_* is the oscillator frequency in pure water) as a function of the glycerin concentration. (**b**) Estimated *R_m_* as a function of the glycerin concentration, *N* = 12.

The relationship between the glycerol concentration, the measured frequency shifts and the estimated resistance was evaluated and reported in Figure 16. It can be seen that, as expected by it being derived from the theory and simulations in the previous section, the effect of using the maximum gain of the amplifier is not a simple offset of the frequency shifts, but it causes an error depending on the working conditions, and as such, it can be hardly compensated. Using the proposed system allows both for reducing the frequency shift error and for adding an accurate measurement of the series resistance, which can be used to assess the viscosity of the interacting liquid.

## 6. Conclusions

This paper showed the feasibility of a novel measurement system based on Quartz Crystal Microbalances, on a Meacham oscillator embedding an amplifier with adjustable gain and on an automatic strategy for gain tuning. It was shown that maintaining the oscillator frequency close to the series resonance frequency of the quartz needs ad-hoc solutions when the quartz operates in-liquid, where the Q-factor is severely reduced. The system uses the optimum gain value to derive the value of the quartz impedance at *f*_0°_, which is a good estimation of the series resistance of the quartz. The simultaneous monitoring of the frequency shift and of the series resistance allows for gaining information on the mechanical load type and distinguishing between the viscous loading and inertial loading of the QCM. The proposed system was tested and verified using a laboratory set-up, but it can be realized with low-cost components exploiting a microcontroller for the implementation of the gain adjustment algorithm and for frequency downshifted signal acquisition and processing, whereas the reference signal can be easily generated with a Direct Digital Synthesis integrated circuit (DDS IC).

## Figures and Tables

**Table 1:** $e_{f}$, the estimated *R_m_, A_vmax_/A_vopt_* and standard deviation of the measured frequency (10 min) in different working conditions. Gly20% is a solution of glycerol in pure water with a 20% concentration.

	*e_f_* = *f_o_@A_vOpt_* − *f_o_@A_vMAX_*	Estimated R_m_ (Ω)	*A_vmax_*/*A_vopt_*	σ_fo_@A_vMAX_ (10 min)	σ_fo_@A_vOpt_ (10 min)
Air	3377 Hz	64	6.66	2.3 Hz	1.3 Hz
Pure water	4390 Hz	237	3.71	20 Hz	8 Hz
Gly20%	4300 Hz	265	3.35	31 Hz	27 Hz

## Data Availability

Data sharing not applicable.
